# What Helps or Hinders Annual Wellness Visits for Detection and Management of Cognitive Impairment Among Older Adults? A Scoping Review Guided by the Consolidated Framework for Implementation Research

**DOI:** 10.3390/nursrep15080295

**Published:** 2025-08-12

**Authors:** Udoka Okpalauwaekwe, Hannah Franks, Yong-Fang Kuo, Mukaila A. Raji, Elise Passy, Huey-Ming Tzeng

**Affiliations:** 1 Department of Academic Family Medicine, College of Medicine, University of Saskatchewan, Saskatoon, SK S7M 3Y5, Canada; udokaokpala.uo@usask.ca; 2 Health System, The University of Texas Medical Branch, Galveston, TX 77555, USA; 3 School of Public and Population Health, The University of Texas Medical Branch, Galveston, TX 77555, USA; yokuo@utmb.edu; 4 John Sealy School of Medicine, The University of Texas Medical Branch, Galveston, TX 77555, USA; muraji@utmb.edu; 5 Alzheimer’s Health Systems Director-Texas, National Division of Health Systems, Alzheimer’s Association, Houston, TX 77087, USA; epassy@alz.org; 6 School of Nursing, The University of Texas Medical Branch, Galveston, TX 77555, USA

**Keywords:** nursing, aged, cognitive impairment, primary care, health care delivery

## Abstract

**Background:** The U.S. Medicare Annual Wellness Visit (AWV) offers a structured opportunity for cognitive screening and personalized prevention planning among older adults. Yet, implementation of AWVs, particularly for individuals with cognitive impairment, remains inconsistent across primary care or other diverse care settings. **Methods:** We conducted a scoping review using the Consolidated Framework for Implementation Research (CFIR) to explore multilevel factors influencing the implementation of the Medicare AWV’s cognitive screening component, with a focus on how these processes support the detection and management of cognitive impairment among older adults. We searched four databases and screened peer-reviewed studies published between 2011 and March 2025. Searches were conducted in Ovid MEDLINE, PubMed, EBSCOhost, and CINAHL databases. The initial search was completed on 3 January 2024 and updated monthly through 30 March 2025. All retrieved citations were imported into EndNote 21, where duplicates were removed. We screened titles and abstracts for relevance using the predefined inclusion criteria. Full-text articles were then reviewed and scored as either relevant (1) or not relevant (0). Discrepancies were resolved through consensus discussions. To assess the methodological quality of the included studies, we used the Joanna Briggs Institute critical appraisal tools appropriate to each study design. These tools evaluate rigor, trustworthiness, relevance, and risk of bias. We extracted the following data from each included study: Author(s), year, title, and journal; Study type and design; Data collection methods and setting; Sample size and population characteristics; Outcome measures; Intervention details (AWV delivery context); and Reported facilitators, barriers, and outcomes related to AWV implementation. The first two authors independently coded and synthesized all relevant data using a table created in Microsoft Excel. The CFIR guided our data analysis, thematizing our findings into facilitators and barriers across its five domains, viz: (1) Intervention Characteristics, (2) Outer Setting, (3) Inner Setting, (4) Characteristics of Individuals, and (5) Implementation Process. **Results:** Among 19 included studies, most used quantitative designs and secondary data. Our CFIR-based synthesis revealed that AWV implementation is shaped by interdependent factors across five domains. Key facilitators included AWV adaptability, Electronic Health Record (EHR) integration, team-based workflows, policy alignment (e.g., Accountable Care Organization participation), and provider confidence. Barriers included vague Centers for Medicare and Medicaid Services (CMS) guidance, limited reimbursement, staffing shortages, workflow misalignment, and provider discomfort with cognitive screening. Implementation strategies were often poorly defined or inconsistently applied. **Conclusions:** Effective AWV delivery for older adults with cognitive impairment requires more than sound policy and intervention design; it demands organizational readiness, structured implementation, and engaged providers. Tailored training, leadership support, and integrated infrastructure are essential. These insights are relevant not only for U.S. Medicare but also for global efforts to integrate dementia-sensitive care into primary health systems. Our study has a few limitations that should be acknowledged. First, our scoping review synthesized findings predominantly from quantitative studies, with only two mixed-method studies and no studies using strictly qualitative methodologies. Second, few studies disaggregated findings by race, ethnicity, or geography, reducing our ability to assess equity-related outcomes. Moreover, few studies provided sufficient detail on the specific cognitive screening instruments used or on the scope and delivery of educational materials for patients and caregivers, limiting generalizability and implementation insights. Third, grey literature and non-peer-reviewed sources were not included. Fourth, although CFIR provided a comprehensive analytic structure, some studies did not explicitly fit in with our implementation frameworks, which required subjective mapping of findings to CFIR domains and may have introduced classification bias. Additionally, although our review did not quantitatively stratify findings by year, we observed that studies from more recent years were more likely to emphasize implementation facilitators (e.g., use of templates, workflow integration), whereas earlier studies often highlighted systemic barriers such as time constraints and provider unfamiliarity with AWV components. Finally, while our review focused specifically on AWV implementation in the United States, we recognize the value of comparative analysis with international contexts. This work was supported by a grant from the National Institute on Aging, National Institutes of Health (Grant No. 1R01AG083102-01; PIs: Tzeng, Kuo, & Raji).

## 1. Introduction

Cognitive impairment, including mild cognitive impairment (MCI) and Alzheimer’s disease, and related dementias (ADRD), affects millions of older adults in the United States, posing challenges to the healthcare system [1]. For these individuals and their caregivers, timely detection and coordinated support are essential [2,3]. However, many care settings, including outpatient, community-based, primary care, and institutional environments, are often under-equipped to proactively manage the complex needs of this population.

To address preventive health and support planning in older adults, the Centers for Medicare and Medicaid Services (CMS) introduced the Annual Wellness Visit (AWV) in 2011, which is a no-cost, structured service for Medicare beneficiaries aged 65 and older [1,4]. AWVs include assessment of physical and cognitive health risks, medication review, personalized prevention planning, and optional advance care planning [4,5]. These visits represent a valuable opportunity for early cognitive screening, patient and caregiver education, and referral to community-based dementia resources [2,6]. AWVs are typically used to conduct brief cognitive assessments targeting memory, attention, orientation, and executive functioning. These screenings, often performed by physicians or nurses, are designed to detect early signs of cognitive impairment and changes in cognition but are not substitutes for comprehensive neuropsychological testing.

In spite of these benefits, AWV implementation for people with MCI/ADRD remains limited and somewhat inconsistent. While nearly 60% of Medicare beneficiaries received 4 to 5 AWVs between 2018 and 2022 [3], there is little understanding of the factors that facilitate or hinder their successful delivery for individuals with cognitive impairment. Additionally, most clinical frameworks (e.g., the Institute for Healthcare Improvement’s 4 Ms framework of an Age-Friendly Health System) emphasize care priorities but do not fully account for the structural, behavioral, and process-level challenges that influence how AWVs are adopted in real-world settings [7]. While our primary interest lies in understanding how AWVs serve individuals with cognitive impairment or at risk of such impairment, we acknowledge that cognitive screening during the AWV is typically performed for all older adult beneficiaries regardless of diagnostic status. Thus, we intentionally included studies examining AWV implementation and cognitive screening more broadly, as these shed light on the structural and process-level determinants that ultimately shape the care of those with or at risk of cognitive decline.

### Study Objective

This scoping review explores multilevel factors influencing the implementation of the Medicare AWV’s cognitive screening component, with a focus on how these processes support the detection and management of cognitive impairment among older adults. We synthesize from published evidence the facilitators and barriers to delivering Medicare’s AWV for cognitive impairment in the United States, using the Consolidated Framework for Implementation Research (CFIR) to guide analysis and synthesis [8,9]. While our primary interest was in implementation within primary care settings, we sought to include studies across a variety of healthcare environments/contexts where AWVs are delivered (including clinics, nursing homes, home-based care, and integrated health systems), recognizing that many older adults receive care in diverse clinical contexts. In this review, we addressed the following research questions:

**Research Question** **1.** *What are the barriers and facilitators to implementing Medicare AWVs with cognitive screening among older adults across diverse care settings, including but not limited to primary care? and*

**Research Question** **2.** *How do these factors interact across different levels of the healthcare system (intervention, individual, organizational, and external)?*

We followed the recommendations of the Preferred Reporting Items for Systematic Reviews and Meta-Analyses Extension for Scoping Reviews (PRISMA-SCR) [10]. This research inquiry will contribute to implementation research by identifying multilevel barriers and enablers that may inform AWV redesign for dementia-sensitive populations.

## 2. Materials and Methods

### 2.1. Study Design and Eligibility Criteria

Our scoping review aimed to identify and synthesize peer-reviewed studies that reported on the facilitators and/or barriers to implementing Medicare’s AWV for older adults with cognitive impairment (including MCI and ADRD) in the United States. We focused specifically on primary data sources (i.e., qualitative, quantitative, and mixed-methods studies) and excluded review articles such as systematic or scoping reviews, opinion pieces, protocols, and other grey literature. Eligible articles met the following criteria:

(a) Published in peer-reviewed journals (original research only),
(b) Focused on AWVs delivered to Medicare beneficiaries in the United States,
(c) Included participants (older adults) with MCI, ADRD, or other related cognitive impairments,
(d) Published between 1 January 2011 and 30 March 2025, and
(e) Written in English.

### 2.2. Protocol and Registration

This study was not registered, and no prior protocol was pre-published before the commencement.

### 2.3. Search Strategy and Data Sources

Working with two medical librarians at the University of Texas Medical Branch Moody Library, we developed a comprehensive search strategy using a combination of keywords and Medical Subject Headings (MeSH) terms related to older adults, AWVs, cognitive impairment, and implementation factors (see Table 1). Searches were conducted in Ovid MEDLINE, PubMed, EBSCOhost, and CINAHL databases. The initial search was completed on 3 January 2024 and updated monthly through 30 March 2025. Reference lists of included articles were also hand-searched using snowballing techniques.

### 2.4. Study Selection

All retrieved citations were imported into EndNote 21 (The EndNote Team, Philadelphia, PA, USA, 2025), where duplicates were removed. The first two authors independently screened titles and abstracts for relevance using the predefined inclusion criteria. Full-text articles (FTAs) were then reviewed and scored as either relevant (1) or not relevant (0). Discrepancies were resolved through consensus discussions. Inter-rater reliability for the final inclusion decisions was high, with a Kappa statistic of 0.876 (standard error of the mean = 0.071, *p* < 0.001), calculated using SPSS software version 28.00, IBM Corp., Armonk, NY, USA.

### 2.5. Data Extraction and Synthesis

We extracted the following data from each included study: Author(s), year, title, and journal; Study type and design; Data collection methods and setting; Sample size and population characteristics; Outcome measures; Intervention details (AWV delivery context); and Reported facilitators, barriers, and outcomes related to AWV implementation.

The first two authors independently coded and synthesized all relevant data using a table created in Microsoft Excel version 16.99.1, Microsoft Corporation, Redmond, WA, USA.

### 2.6. Data Analysis

The Consolidated Framework for Implementation Research (CFIR) [8,9] guided our data analysis, thematizing our findings into facilitators and barriers across its five domains, viz: (1) Intervention Characteristics, (2) Outer Setting, (3) Inner Setting, (4) Characteristics of Individuals, and (5) Implementation Process.

#### Analytic Framework

The CFIR is a widely adopted meta-theoretical framework that helps identify and categorize multilevel factors affecting the implementation of health interventions [8,9]. It generally provides a structured approach for analyzing why interventions succeed or fail in real-world settings by organizing data across five key domains, namely:

(a) Intervention characteristics: The intervention characteristics domain refers to the features of the intervention itself that influence how easily it can be implemented, adopted, and sustained in a real-world setting. This includes both perceived and actual attributes of the intervention. In the context of our study, it may include the attributes of the AWV itself (e.g., complexity, adaptability).
(b) Outer setting: The outer setting domain refers to the external influences that impact the implementation of an intervention within an organization or system. This domain focuses on how environmental and stakeholder factors outside the implementing organization shape success or failure. In the context of this study, it includes external influences such as patient needs, policy incentives, and resource availability. Patient needs capture factors, including but not limited to cognitive status (e.g., degree of impairment), comorbid conditions, mobility limitations, and sociodemographic considerations, including rurality, race/ethnicity, and digital literacy, as influenced by AWV uptake and delivery. Policy incentives could include federal or payer-level enablers such as clear billing codes, Medicare Advantage program bonuses, Accountable Care Organization (ACO) alignment, and participation in advanced care models that reward AWV completion. Availability of resources would include clinic-level infrastructure such as the presence of EHR prompts or templates, access to trained staff or cognitive screening tools, interprofessional team support, and leadership buy-in for preventive services.
(c) Inner setting: The Inner Setting focuses on the internal context in which the intervention is implemented. This includes the organization’s structure, culture, workflow, and readiness for change.
(d) Characteristics of individuals: This domain refers to the people involved in implementation, particularly their beliefs, knowledge, self-efficacy, and the attitudes of those involved in delivering the AWV (e.g., providers).
(e) Implementation process: This focuses on the actual activities and strategies used to roll out and sustain the intervention, which includes specific strategies, engagement efforts, and steps taken to operationalize AWV delivery.

Using CFIR enabled us to examine not only what facilitators or barriers were reported, but also where they occurred within the healthcare system and how they influenced the implementation of AWVs for older adults with cognitive impairment [8,9].

### 2.7. Data Synthesis

Following analysis, we mapped extracted findings from the included studies onto the CFIR domains. Barriers and facilitators were coded into thematic categories, and their alignment with CFIR helped organize the results across systemic, organizational, interpersonal, and individual levels. This approach provided a comprehensive interpretive lens to identify leverage points and gaps in the current implementation of AWVs for Medicare beneficiaries with MCI/ADRD.

### 2.8. Reflexivity, Rigor, and Trustworthiness of Findings

To ensure the trustworthiness of findings, we applied an inductive-deductive (abductive) approach guided by the CFIR constructs to identify themes that fall into facilitators or barriers to implementing AWVs. We met weekly via video calls to review codes, reconcile discrepancies via consensus, and iteratively refine themes based on the CFIR domains. Emerging patterns were discussed until analytic consensus or thematic saturation was reached.

### 2.9. Quality Appraisal

To assess the methodological quality of the included studies, we used the Joanna Briggs Institute critical appraisal tools appropriate to each study design (e.g., cross-sectional, retrospective, prospective, randomized controlled trials). These tools evaluate rigor, trustworthiness, relevance, and risk of bias [11]. Results of the quality appraisal are presented in Appendix A, Appendix B, Appendix C and Appendix D.

## 3. Results

### 3.1. Selection of Evidence Sources

**Figure 1:**
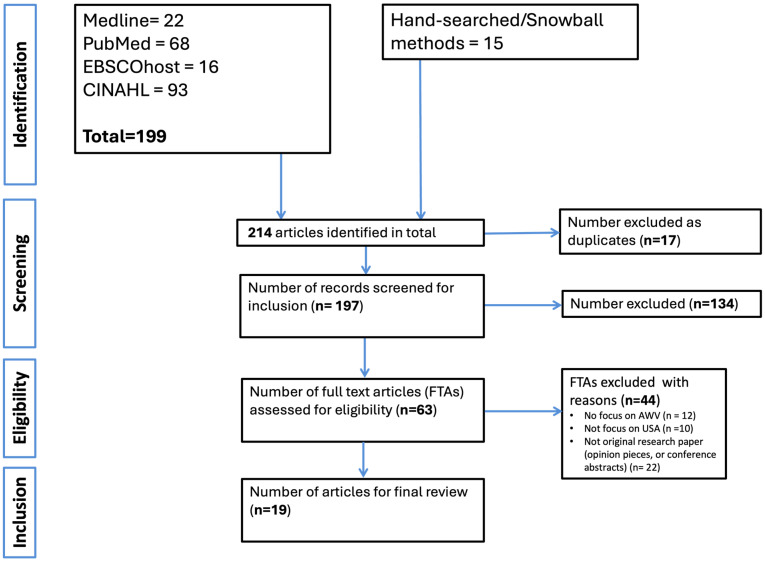
Flowchart of identification of studies.

We identified 214 records through searches across four electronic databases and manual hand-searching. After removing 17 duplicates, 197 titles and abstracts were screened. Of these, 63 FTAs were reviewed in detail. Following full-text screening, 19 studies met the inclusion criteria and were included in the final synthesis of this review. Common reasons for exclusion at the FTA stage included lack of specific focus on AWVs (n = 12), studies not conducted in the U.S. context (n = 10), or the article not being original research (e.g., opinion pieces or conference abstracts) (n = 22). The article selection process is illustrated in the PRISMA flow diagram (Figure 1) below.

### 3.2. Characteristics of Evidence Sources

The 19 included studies demonstrated a predominantly quantitative focus, with 16 (84.2%) employing quantitative designs and only three (15.7%) utilizing mixed methods. No qualitative-only studies were identified. Most studies (14/19; 73.7%) were published between 2021 and 2025, reflecting a growing interest in AWV implementation in recent years. Most studies (15/19; 78.9%) used secondary data sources, such as Medicare claims or administrative registry data, while four (21.1%) used primary data collection. Twelve (63.1%) of the included studies had a national scope, while the remaining were conducted in specific states across the United States, including Maryland, Virginia, Pennsylvania, Missouri, Texas, and Hawai’i. A summary of the general and methodological descriptive characteristics is presented in Table 2.

### 3.3. Results of Individual Sources of Evidence

Across all included studies, AWVs were conducted by a range of personnel, including physicians, nurse practitioners/nurses, and (in team-based or resource-constrained settings) medical assistants or care coordinators. The specific provider roles often varied by setting and organizational capacity, with some clinics leveraging interdisciplinary teams to deliver components of the visit. A summary of individual sources of evidence is presented in Table 3. Each article was included based on its relevance to one or more of the study’s research questions.

### 3.4. Synthesis of Findings

Our findings were thematically organized using CFIR [9], mapping barriers and facilitators across five domains: Intervention characteristics, outer setting, inner setting, characteristics of individuals, and implementation process (see Table 4).

### 3.5. Facilitators to Implementing AWVs

We summarize the facilitators to implementing Medicare AWVs for older adults with cognitive impairment in Table 4. Using the CFIR framework, we categorized findings across five domains.

#### 3.5.1. Intervention Characteristics

The domain of intervention characteristics looked at the attributes of the AWV specifically, its complexity, adaptability, design quality, and relative advantage compared to standard practices. Several included studies identified how these inherent features of the AWV either facilitated or hindered its delivery to older adults with cognitive impairment. A key facilitator to implementing Medicare AWVs was their perceived adaptability within existing clinical workflows, particularly in interdisciplinary or structured care environments. Integration of customized tools like the PROMIS Cognitive Function Screener (PRO-CS) into electronic health records (EHRs) enhanced provider engagement and communication around cognitive health [17]. Similarly, embedding AWVs in nursing home settings through standardized protocols supported alignment between intervention design and the complex needs of older adults [22]. Across included studies, AWVs were seen as offering a relative advantage over routine or ad hoc visits as they provided structured opportunities for early cognitive screening, patient education, medication reconciliation, and dementia risk management [18,29]. A few studies explicitly described the use of structured or formal screening tools, while others referenced cognitive assessments more generally or contrasted them with informal clinician queries. For example, Harrison et al. [17] examined the implementation of the PRO-CS (Patient-Reported Outcome Cognitive Screener) and its integration into AWV workflows. JaKa et al. [19] evaluated a dementia-friendly cognitive screening tool embedded in family medicine clinics, emphasizing its acceptability, tool clarity, and feasibility. Liu et al. [23] and Jacobson et al. [14] distinguished between formal cognitive testing and informal clinician questions, with Liu et al. [23] noting that only a fraction of beneficiaries received structured screening. Powell et al. [27] reported on the use of structured cognitive assessment prompts as part of shared decision-making documentation. While Hamer et al. [30] referred to cognitive screening and workflow templates, specific tools were not named (see Table 3 for more examples of cognitive screening tools). Similarly, educational components for patients and caregivers were inconsistently reported. JaKa et al. [19] highlighted the role of patient-provider trust and communication clarity in facilitating understanding of cognitive screening results. Smith et al. [28] described the SHARING Choices intervention, which included mailed materials, checklists, and patient portal access to prepare older adults and caregivers for AWV discussions. However, most studies did not describe the scope, format, or use of standardized educational templates, indicating a need for more consistent educational practices (see Table 3 for more). Clinic-level infrastructure, such as effective EHR integration, interdisciplinary care teams, and standardized workflows, was associated with improved AWV implementation. See more in Table 4.

#### 3.5.2. Outer Setting

In the outer setting domain, we explored the external influences affecting AWV implementation, such as policies, patient needs and resources, and community-level factors. Several outer setting elements identified in our review emerged as enablers to delivering AWVs to older adults with cognitive impairment.

A consistent facilitator across studies was the alignment of policy and financial incentives with AWV implementation. Participation in federal initiatives such as the Comprehensive Primary Care Plus (CPC+) program and other value-based payment models, including Accountable Care Organizations and Medicare Advantage Plans, was associated with higher AWV uptake and expanded delivery of preventive services. These models promoted AWV adoption by tying reimbursement to preventive care performance metrics and enabling bundled care approaches [14,18,20,23,29].

#### 3.5.3. Inner Setting

The inner setting domain in CFIR captures the structural, cultural, and resource-related conditions within clinical organizations that shape how AWVs are implemented. Key elements include organizational readiness, communication structures, leadership engagement, and available infrastructure [9]. Across several studies, organizational support (particularly from leadership) and a culture of continuous quality improvement were identified as critical enablers of AWV delivery. Clinics that had dedicated champions (e.g., geriatric-focused providers or nurse leads) and engaged multidisciplinary teams were more likely to embed AWVs into routine workflows effectively [22,27]. Another facilitator was the integration of AWV templates and cognitive screening tools into EHRs [17]. Similarly, Hamer et al. (2023) noted that clinics with EHR prompts, dedicated AWV champions, and organizational commitment to preventive care delivery reported more consistent implementation of AWVs, especially when aligned with value-based care models [30]. This technological alignment improved documentation processes and enabled standardized delivery of AWV components. For instance, the incorporation of the PRO-CS screener into EHR systems significantly enhanced both provider engagement and the frequency of cognitive assessments [17]. Additionally, the assignment of staff in specific roles (such as nurses, social workers, or care coordinators) to manage AWV-related tasks improved operational efficiency. In several cases, embedding these responsibilities within existing workflows, rather than layering them as add-ons, contributed to the long-term feasibility and sustainability of AWV integration in primary care [25,29].

#### 3.5.4. Characteristics of Individuals

This CFIR domain examines the knowledge, attitudes, confidence, and beliefs of individuals responsible for implementing AWVs, including primary care providers, nurses, and allied health staff. These personal and professional attributes play a critical role in determining whether AWVs (especially those involving cognitive screening) are delivered consistently and effectively. Across included studies, one key facilitator was providers’ belief in the clinical value of AWVs for early dementia detection and proactive care planning. Clinicians who perceived AWVs as beneficial were more likely to implement them routinely and with fidelity [21,26]. This confidence was further bolstered by training and exposure to standardized tools, such as the PRO-CS, which enhanced provider comfort in addressing cognitive concerns during visits [17], the Mini-Cog [19], and the General Practitioner Assessment of Cognition (GPCOG) [19]. These tools were typically embedded within electronic health record (EHR) templates or prompted via workflow alerts.

Providers involved in cognitive screening during AWVs included physicians, nurse practitioners/nurses, and, in some cases, medical assistants or care coordinators working within interdisciplinary teams [19,25,28]. Training and exposure to these tools varied across settings. Some studies described formal training modules or workshops offered as part of dementia-friendly practice initiatives [19], while others relied on informal EHR prompts, peer champions, or provider self-directed learning [17,30]. Notably, JaKa et al. [19] highlighted the importance of clear tool instructions and structured workflows in supporting implementation fidelity. Harrison et al. [17] also reported that language framing during tool administration influenced provider and patient engagement. Similarly, Harrison et al. [17] reported that communication style also influenced uptake. When cognitive assessments were framed using strengths-based language (emphasizing abilities rather than deficits), providers reported more productive interactions and patients were more receptive to participation [17]. These findings highlight the importance of individual readiness and interpersonal communication in shaping AWV implementation. Additionally, intrinsic motivation and professional identity among healthcare providers (particularly physicians and nurse practitioners) played an important role in AWV delivery. Providers with a geriatric focus or prior experience managing ADRD were more likely to complete AWVs thoroughly and make appropriate referrals [23,25].

#### 3.5.5. Implementation Process

This CFIR domain focuses on the strategies and actions used to integrate AWVs into clinical practice, including planning, execution, monitoring, and evaluation. Key elements here include team engagement, workflow alignment, and ongoing quality improvement. Team engagement was highlighted as a facilitator to the successful implementation of AWVs, particularly when interdisciplinary team members (including physicians, nurse practitioners/nurses, medical assistants, and care coordinators) had clearly defined roles and communication was streamlined. In several studies [13,17,22,27], team-based workflows enhanced the efficiency of cognitive screening, documentation, and follow-up planning. While AWVs are typically conducted annually, some included studies [20,22,27] described settings where structured follow-up visits or repeat screenings were incorporated as part of dementia care pathways. Several scoping studies identified structured implementation approaches as critical facilitators. Embedding standardized templates, checklists, and prompts into EHR systems helped guide providers during AWVs, ensuring consistent completion of key components like cognitive screening [17,23]. Staff training and pre-visit planning were also instrumental. Clinics that prepared their teams to review patient records, gather baseline data, and initiate conversations prior to visits experienced more efficient workflows and improved patient engagement [27,28]. Interdisciplinary models, including the use of care coordinators or clinical champions, further enhanced implementation. Additionally, sites that used phased rollouts and iterative quality improvement methods reported greater adoption and refinement over time [27]. Clear protocols, defined follow-up pathways, and leadership support were common features of high-performing implementations [24,28].

### 3.6. Barriers to Implementing AWVs

As with the facilitators, we thematized barriers or challenges to implementing AWVs using the CFIR as a framework for inductive-deductive analysis. See Table 4 for more information.

#### 3.6.1. Interventional Characteristics

Despite its preventive intent, several studies highlighted limitations in the implementation and clinical utility of the AWV model. A key barrier was the complexity and perceived burden of delivering all AWV components within standard visit durations, particularly cognitive assessments, which providers often struggled to complete [12,27]. The ambiguity surrounding the cognitive screening requirement was also cited as a challenge [19]. Additionally, the optional nature of cognitive assessments and the lack of standardized guidance on follow-up pathways contributed to inconsistent detection and management of cognitive concerns [23]. Notably, while the AWV was designed to enhance early identification of cognitive decline, its real-world clinical impact was mixed. Fowler et al. [12] found no significant differences in dementia diagnoses or prescriptions of dementia-related medications between AWV and non-AWV groups [12]. Moreover, some studies pointed to challenges in interpreting patient-reported tools like PRO-CS, noting limited clarity on how results should inform clinical decision-making [17].

#### 3.6.2. Outer Setting

Several outer setting barriers hindered the effective implementation of AWVs, particularly among older adults with cognitive impairment. Chief among these were systemic and policy-level constraints. Studies frequently cited vague guidance from CMS regarding what constitutes an acceptable cognitive assessment, along with limited reimbursement for such tools. These ambiguities led to inconsistent documentation, billing, and, in some cases, omission of cognitive components entirely [19]. Socioeconomic and geographic inequities also emerged as critical challenges. Uptake of AWVs was significantly lower among underserved groups (e.g., rural residents, racially diverse populations, and dual-eligible beneficiaries) due to structural barriers such as limited transportation, poor broadband access for telehealth visits, and low availability of culturally appropriate care [15,16,26,27]. Furthermore, many patients in disadvantaged communities lacked awareness of AWVs or deprioritized them in the face of more pressing health and social needs [12,20]. At the individual level, patient-related challenges such as mobility impairments, multimorbidity, and dementia-related limitations often made participation in AWVs difficult. Those with early-stage cognitive decline or multiple chronic conditions faced practical barriers to attending or completing the visit, further compounding disparities in access and continuity of care [22]. These studies acknowledged external pressures affecting AWV implementation in underserved communities, including limited provider availability in rural regions, technological and transportation barriers, and disparities in health literacy. These barriers disproportionately affect ethnic minorities and low-income older adults, many of whom face intersecting access challenges due to historical underinvestment in their communities.

#### 3.6.3. Inner Setting

Multiple studies highlighted structural and operational constraints within clinical settings that impeded the consistent implementation of AWVs. Staffing shortages, fragmented documentation systems, and competing clinical demands frequently disrupted AWV workflows [13]. Clinics often struggled to align AWVs with routine visit structures due to the time-intensive nature of the visit, the need for significant paperwork, or the requirement to schedule additional appointments [24]. Another recurring barrier was insufficient training and staff awareness, particularly around cognitive assessment components. In settings lacking clear internal protocols or standardized workflows, AWVs were delivered inconsistently across providers [23]. Hamer et al. [30] further emphasized structural and cultural challenges, such as workflow interruptions, limited billing clarity around cognitive assessments, and low perceived value among some providers. In clinics lacking strong leadership engagement or clear EHR support, AWVs were frequently viewed as burdensome or optional amidst competing demands [30]. Furthermore, organizational priorities were not always aligned with AWV delivery. In clinics that focused more on acute or high-volume care, AWVs were deprioritized despite policy incentives. As a result, limited time and administrative resources were allocated for their implementation [19].

#### 3.6.4. Characteristics of Individuals

A few studies highlighted provider-level barriers to delivering cognitive screening during AWVs. A consistent challenge was clinicians’ limited confidence or familiarity with the cognitive assessment process, ranging from uncertainty about which tools to use, how to interpret results, or what follow-up actions to take [12,17,24]. This uncertainty often led to hesitation or omission of the cognitive component altogether. Discomfort initiating conversations about cognitive decline was another common barrier. Some providers feared causing distress or felt ill-equipped to navigate these sensitive discussions, especially in brief visits or when patient-provider rapport was limited [27]. Additionally, competing clinical demands influenced provider behavior. In high-pressure settings, AWVs were often deprioritized in favor of acute care needs, even when eligible patients were willing to participate [20].

#### 3.6.5. Implementation Process

A recurring barrier across studies was the absence of a clearly defined and standardized implementation process for AWVs. Many clinics lacked structured workflows, designated roles, or clear expectations for who should initiate and complete AWV components, especially cognitive assessments [12,15,21]. This gap in operational planning often led to inconsistent delivery or omission of key elements. Inadequate training and onboarding further compounded these challenges. A lack of routine performance monitoring also limited quality improvement. Few practices systematically tracked AWV completion rates or dementia-related quality indicators, weakening accountability and reducing momentum for programmatic refinement [16,20]. Finally, insufficient leadership engagement hindered effective integration. In practices where AWVs were not championed by leadership or aligned with organizational goals, they were often viewed by staff as low-priority or burdensome additions to already demanding clinical workflows [13,18,29].

### 3.7. Cross-Domain Patterns Identified by CFIR Domains

In analyzing the factors that shape AWV implementation for older adults with cognitive impairment, we observed several interrelated patterns across CFIR domains. While no causal inferences can be made due to the nature of our scoping review, the data revealed recurring interdependencies that shaped implementation successes. In particular, policy-level incentives from the Outer Setting (e.g., reimbursement through CMS) were frequently cited but rarely sufficient on their own. Their effectiveness depended on supporting structures within the Inner Setting, such as adequate staffing levels, integrated EHR prompts, and organizational readiness. Likewise, even well-designed Intervention Characteristics (e.g., standardized cognitive screening tools) require enabling Implementation Processes (such as training modules, structured workflows, and leadership buy-in) to be effectively and consistently adopted. These processes also had to be reinforced by provider-level Characteristics of Individuals, including clinical confidence, geriatric focus, and belief in the value of AWVs. Notably, breakdowns in one domain often undermined progress in others. For example, some studies described promising reimbursement models and toolkits that failed to produce uptake due to poor onboarding processes or limited team engagement. These examples highlight the complex web of reinforcing dynamics at play across system levels.

**Figure 2:**
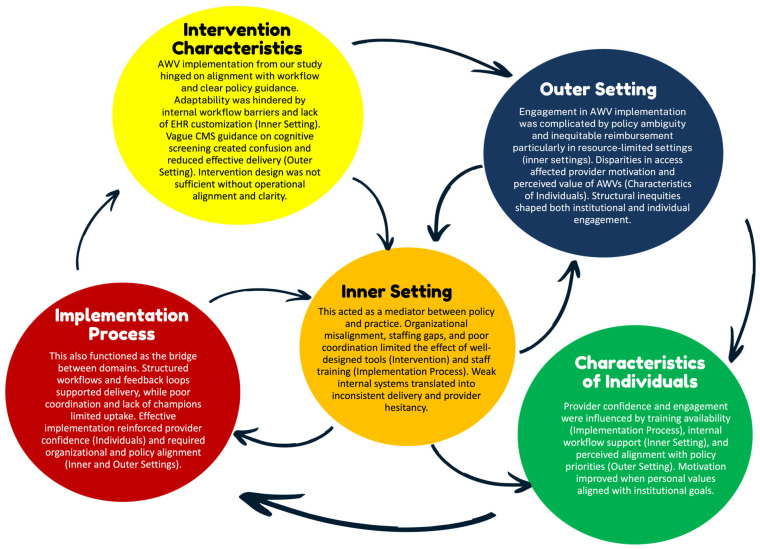
Cross-Domain Patterns influencing AWV implementation. Notes: This figure illustrates key interrelationships across CFIR domains based on patterns observed in the included studies. Arrows reflect conceptual influence rather than tested causal pathways, highlighting how multiple domains must align to support the successful implementation of Annual Wellness Visits with cognitive screening. Abbreviations: AWV: Annual Wellness Visit; CFIR: Consolidated Framework for Implementation Research; CMS: Centers for Medicare and Medicaid Services; EHR: Electronic Health Record.

To synthesize these patterns, we mapped the most prominent interrelationships in Figure 2, illustrating how structural, behavioral, and contextual factors interact. While these connections do not imply causality, they offer conceptual insight into potential leverage points for strengthening AWV implementation. Future studies using realist evaluation or systems theory approaches may build upon these relationships to better assess mechanisms and outcomes.

## 4. Discussion

This scoping review used CFIR [9] to examine the implementation of Medicare’s AWV with cognitive assessment in the United States. Our findings underscore the fact that successful use of AWVs is shaped by a web of interacting influences across all CFIR domains. No single factor explains success or failure; instead, the effectiveness of AWVs hinges on the alignment of intervention design, system infrastructure, provider readiness, and policy context [9,18,22,23,27]. These lessons hold valuable implications for the aging community in the United States as well as in other jurisdictions where the integration of preventive cognitive screening into primary care is gaining relevance amidst widening primary care access gaps [31,32,33]. Although several studies examined the use of cognitive screening during AWVs, there was notable variability in the tools employed and limited reporting on educational support for patients and caregivers. Only a few studies, like Harrison et al. [17] and JaKa et al. [19], described specific screening tools integrated into workflows. Similarly, while interventions like SHARING Choices incorporated educational materials and structured prompts, the broader literature lacked detail on the format, delivery, and standardization of cognitive health education. These gaps underscore an opportunity to improve clarity and consistency in both screening practices and patient-facing communication during AWVs.

Facilitators were most prominent in the domains of Intervention Characteristics and Implementation Process [17,18,22,29]. The AWV’s adaptability (especially when structured tools like PRO-CS and shared decision-making aids were embedded in workflows) supported dementia-sensitive conversations and enhanced provider confidence [18,22]. Clinics with established EHR templates, role-specific task delegation, and pre-visit planning protocols reported smoother implementation [22,27]. When supported by leadership, quality improvement culture, and interprofessional teams, AWVs were more likely to be adopted and sustained [22,27]. Outer Setting incentives, such as participation in Medicare Advantage or ACOs, further encouraged uptake. Financial alignment and bundled care models facilitated AWV integration, especially when paired with relational continuity between patients and their primary care teams [14,18,20,23,29]. However, health equity emerged as an implicit but under-explored dimension across the included studies. Although reimbursement policies and workforce support are discussed, few studies have explicitly examined how systemic inequities (e.g., structural racism, digital divides, and geographic maldistribution of services) influence the reach and effectiveness of AWV implementation. Our findings underscore a need for future implementation research to center equity frameworks and better understand how AWVs can be adapted to meet the needs of marginalized populations. Although our initial focus was on primary care, the inclusion of studies from a variety of care settings (including specialty clinics, large health systems, and long-term care facilities) reflects the complex and distributed nature of AWV implementation across the U.S. healthcare system. This broader scope offers valuable insights into how organizational structure, staffing, and system-level resources shape implementation processes and outcomes. At the same time, we recognize that this diversity limits our ability to draw setting-specific conclusions. Future reviews may consider stratifying results by care setting to illuminate how AWV implementation unfolds in resource-constrained primary care environments compared to more integrated or specialized systems.

These findings echo broader evidence that effective delivery of preventive care in older adults often depends on stable, team-based infrastructure [34,35,36,37]. At the individual level, confidence, familiarity with screening tools, and intrinsic motivation played central roles. Providers with prior experience in dementia care, or those working in geriatric-focused settings, were more likely to complete AWVs with fidelity. Strengths-based communication and culturally-attuned framing also improved provider-patient interaction, suggesting a need for training in relational aspects of cognitive care. The variation in how AWV-related facilitators and barriers manifested across different care settings is noteworthy. While our review initially emphasized primary care, we found that implementation dynamics were shaped by the broader organizational context. For instance, tools like the PROMIS Cognitive Function Screener and structured multidisciplinary teams were more commonly reported in large healthcare systems and specialty geriatric settings, where resources, staffing, and operational protocols tended to be more robust. In contrast, primary care environments (particularly smaller or under-resourced practices) were more likely to report challenges such as workflow fragmentation, lack of cognitive screening integration, and inconsistent team structures. These discrepancies suggest an opportunity for cross-setting learning. Health systems with strong AWV infrastructure may serve as “organizational mentors,” offering models and technical support for embedding dementia-sensitive workflows in primary care. Facilitating such mentorship and adaptation may be key to expanding the reach and fidelity of cognitive screening across the continuum of care in the US.

Despite its promise, the AWV remains inconsistently delivered, particularly for patients with cognitive impairment. Barriers cut across Inner and Outer Settings, with compounding effects. Many clinics, especially those in rural or under-resourced areas, faced staffing shortages, fragmented documentation systems, and misaligned incentives that deprioritized preventive visits in favor of acute care [23,24,29,38]. CMS billing ambiguity, inadequate reimbursement for cognitive screening, and unclear guidance on structured assessments further contributed to confusion and inconsistent implementation [14,39]. At the Intervention level, questions about clinical utility remain. Studies found no significant differences in dementia diagnoses or management between AWV and non-AWV groups, which raises questions about whether current designs effectively trigger follow-up care [12,16,18,22]. Additionally, implementation processes were often poorly defined, lacking standardized workflows, designated leads, or feedback mechanisms. Without active management or embedded accountability structures, AWVs were perceived by frontline staff as optional or burdensome [13,18,29,40]. In addition, Hamer et al. [30] highlighted structural and cultural challenges in AWV implementation, including workflow interruptions, lack of clarity around billing for cognitive assessments, and variable perceived value among providers. Clinics without strong leadership engagement or EHR support struggled to prioritize AWVs, while frontline staff often viewed them as optional amidst competing demands [30]. Provider hesitation also emerged as a barrier. Some clinicians lacked confidence in interpreting cognitive screening results or initiating conversations about dementia, particularly in time-limited visits [17,27]. Competing clinical priorities and discomfort with sensitive topics further contributed to low uptake, even when patients were eligible and willing [20,40]. For example, Fowler et al. [12] reported no significant relationship between AWV completion and cognitive impairment diagnoses. This finding may reflect several contextual factors, including variability in how AWVs were conducted, the use (or non-use) of validated cognitive screening tools, and documentation practices across settings. It also underscores the importance of implementation fidelity and provider engagement in shaping the effectiveness of AWVs as a screening mechanism. Such inconsistencies in outcomes highlight the need for more standardized delivery models and clearer outcome definitions in future studies.

### 4.1. Practical Implications

The findings from our review offer practical guidance for strengthening AWV implementation in primary care. Strategies that enhance AWV uptake, such as structured templates, shared decision-making tools, workflow standardization, team-based delivery, and financial alignment—can inform not only AWV redesign but also broader dementia care models. For instance, the Guiding an Improved Dementia Experience (GUIDE) Model, recently launched by CMS, relies on the timely identification and ongoing engagement of patients with MCI or dementia [41]. Implementation strategies identified in this review may be transferable to GUIDE or similar dementia-focused programs, supporting improved coordination, early detection, and caregiver support. As such, AWV-related quality improvement efforts may serve as a stepping stone toward broader transformation of cognitive care in aging populations.

The findings of this review point to several strategies for optimizing the implementation of AWVs for older adults with cognitive impairment. First, embedding cognitive screening into standardized clinical workflows (supported by EHR-integrated tools, role-specific delegation, and pre-visit planning) can improve delivery consistency and reduce provider burden [22,27,29]. Second, targeted training in dementia-sensitive communication and interpretation of cognitive assessment tools is essential to enhance provider confidence and reduce hesitation [17,19]. Organizational leadership must actively champion AWVs as core components of preventive care, aligning them with broader quality improvement and care coordination goals [24,27]. At the system level, clarifying CMS billing guidance and enhancing reimbursement for cognitive assessments may address financial disincentives that currently limit uptake [14,18,20]. These insights are not only relevant to U.S. Medicare settings but also have high translatability to other countries, where primary care systems face similar challenges, including access issues, provider shortages, and the need to integrate dementia care into routine practice [31,32,33]. Although our scoping review did not isolate findings by provider role, many of the facilitators identified (such as structured workflows, cognitive screening protocols, and team-based planning) fall within the scope of nursing practice in outpatient settings. Nurses, including care coordinators and nurse practitioners, are often at the frontline of Annual Wellness Visit delivery and follow-up. Their involvement in workflow design, patient education, and cognitive screening makes them well-positioned to champion and spread effective implementation practices, especially in under-resourced settings. Integrating nurses into implementation planning and leadership may be critical for improving AWV uptake and fidelity, particularly where interprofessional collaboration is central to success.

### 4.2. Study Strengths and Limitations

Our study may be the first known scoping review to systematically apply the CFIR framework to examine the implementation of AWVs for older adults with cognitive impairment in the U.S. Using a structured implementation science lens allowed us to identify cross-domain influences, offering a holistic understanding of the barriers and enablers affecting AWV uptake. While causality cannot be inferred from scoping reviews, the patterns observed suggest key areas where structural factors (e.g., payment models, training supports) consistently shaped implementation behaviors. The review is timely, given the growing interest in dementia-sensitive primary care, and is directly translatable to other jurisdictions. Additionally, our synthesis integrates practical, policy-relevant insights that can inform AWV redesign and implementation planning.

However, a few limitations should be acknowledged. First, our scoping review synthesized findings predominantly from quantitative studies, with only two mixed-method studies [19,24] and no studies using strictly qualitative methodologies. As such, important experiential and behavioral drivers at the patient and caregiver level, as well as nuanced, individual-level insights (such as clinicians’ emotional responses, communication barriers, and patients’ lived experiences), are often better captured through qualitative inquiry. Although our review did not critically appraise the statistical validity of included quantitative studies (in line with scoping review guidance), variability in study design, outcome definitions, and analytic approaches may have contributed to mixed or null findings. These inconsistencies underscore the importance of cautious interpretation when comparing across studies.

Second, few studies disaggregated findings by race, ethnicity, or geography, reducing our ability to assess equity-related outcomes. Moreover, few studies provided sufficient detail on the specific cognitive screening instruments used or on the scope and delivery of educational materials for patients and caregivers, limiting generalizability and implementation insights.

Third, grey literature and non-peer-reviewed sources were not included, which may have excluded relevant program evaluations or quality improvement reports from health systems.

Fourth, although CFIR provided a comprehensive analytic structure, some studies did not explicitly fit in with our implementation frameworks, which required subjective mapping of findings to CFIR domains and may have introduced classification bias. Additionally, although our review did not quantitatively stratify findings by year, we observed that studies from more recent years were more likely to emphasize implementation facilitators (e.g., use of templates, workflow integration), whereas earlier studies often highlighted systemic barriers such as time constraints and provider unfamiliarity with AWV components. This may reflect an evolving understanding and optimization of AWV processes over time and may be considered a limitation in our study. Nonetheless, while our review was motivated by concerns about AWV implementation for individuals with cognitive impairment, we observed that most included studies addressed AWV delivery and cognitive screening more broadly among older adults. We view this as a strength, reflecting the real-world implementation context of AWVs, where cognitive impairment is often underdiagnosed and screening serves as a key gateway to recognition and care. As such, insights about structural barriers, workflow integration, and provider readiness to conduct cognitive screening have high relevance for improving care among patients with undetected or early-stage cognitive impairment.

Finally, while our review focused specifically on AWV implementation in the United States, we recognize the value of comparative analysis with international contexts. Although outside the scope of this scoping review, future research could examine how analogous initiatives (such as age-friendly health systems and cognitive screening practices) are implemented in Canada and European countries. Such comparisons may illuminate transferable strategies or context-specific barriers, thereby enhancing the global applicability of insights related to AWV implementation. We view this as an important direction for follow-up work, particularly as many countries face similar challenges related to primary care access, dementia screening integration, and the delivery of preventive services in aging populations.

## 5. Conclusions

This scoping review highlights the fact that the successful implementation of AWVs for older adults with cognitive impairment is shaped by a complex interplay of factors across policy, organizational infrastructure, provider attitudes, and implementation processes. While AWVs offer a promising platform for early dementia detection and proactive care planning, their impact remains constrained by fragmented workflows, inconsistent training, underdeveloped implementation strategies, and inequities in access. Practical solutions should go beyond policy and focus more on team-based delivery models, clinical infrastructure, and provider engagement. Greater clarity in CMS guidance, integration of culturally tailored screening tools, and investment in health information technology and workforce capacity could be critical next steps to move this tool forward.

## Figures and Tables

**Table 1:** Summary of keywords and Medical Subject Headings (MeSH) search terms used in Ovid MEDLINE, PubMed, EBSCOhost, and CINAHL.

**Population** 1. Aged/OR Aged, 80 and over/OR Aging/ 2. (older adj2 adult*) OR elder* OR aging OR senior* OR geriatric* OR aged OR “age 65 and over” OR “community-dwelling seniors” OR “older persons”.ti,ab **Condition** 3. Cognition Disorders/OR Alzheimer Disease/OR Dementia/OR Cognitive Dysfunction.ti.ab 4. (cognit* adj2 impair*) OR dementia OR Alzheimer* OR “mild cognitive impairment” OR MCI OR ADRD.ti,ab **Setting** 5. Preventive Health Services/OR Health Services for the Aged/OR Primary Health Care/ 6. (annual adj2 wellness adj2 visit*) OR AWV.ti,ab **Implementation/Geography** 7. Medicare/OR Centers for Medicare and Medicaid Services (USA only)/ 8. Medicare OR CMS OR “Medicare Advantage” OR “Medicare beneficiaries”.ti,ab 9. Program Evaluation/OR Implementation Science/OR Quality Improvement/ 10. (facilitat* OR barrier* OR implement* OR uptake OR adoption OR dissemination OR delivery).ti,ab 11. 1 OR 2 12. 3 OR 4 OR 5 OR 6 13. 7 OR 8 OR 9 OR 10 14. 11 AND 12 AND 13

Abbreviation: CMS: Centers for Medicare and Medicaid Services.

**Table 2:** General and methodological characteristics of included studies (n = 19).

Publication Year	n (%)	Article Citation
2011–2015	0 (0.0)	--
2016–2020	5 (26.3)	[12,13,14,15,16]
2021–2025	14 (73.7)	[17,18,19,20,21,22,23,24,25,26,27,28,29,30]
Location *		
Baltimore	1 (5.3)	[28]
Maryland	1 (5.3)	[25]
Pennsylvania	1 (5.3)	[17]
Virginia	1 (5.3)	[25]
Washington D.C.	1 (5.3)	[25]
Hawai’i	1 (5.3)	[24]
Midwest region	1 (5.3)	[19]
Missouri	1 (5.3)	[22]
Texas	1 (5.3)	[29]
Nationwide	13 (68.4)	[12,13,14,15,16,18,20,21,23,26,27,30]
**Data collection period (Study timeframe)**		
2020	1 (5.3)	[26]
2003–2014	1 (5.3)	[21]
2010–2014	2 (10.5)	[12,13]
2013–2017	1 (5.3)	[16]
2014–2018	1 (5.3)	[29]
2015–2018	1 (5.3)	[30]
2017–2022	1 (5.3)	[27]
Not reported (NR)	11 (57.8)	[14,15,17,18,19,20,22,23,24,25,28]
**Study type**		
Quantitative	16 (84.2)	[12,13,14,15,16,17,18,20,21,22,23,25,26,27,28,29]
Qualitative	0 (0.0)	--
Mixed Methods Research	3 (15.7)	[19,24,30]
**Data collection type**		
Primary data	4 (21.1)	[19,23,24,28]
Secondary data (Data Registry/admin data)	15 (78.9)	[12,13,14,15,16,17,18,20,21,22,25,26,27,29,30]

* Multiple locations may overlap.

**Table 3:** Characteristics of included studies (n = 19).

Author (Citation)	Study Objective	Methodology and Methods	Outcome Measure/Intervention	Relevant Findings	
				Facilitators	Barriers
Fowler et al., 2018 [12]	To assess the effect of the AWV on cognitive impairment detection and associated lab testing or treatment patterns.	Study type: Quantitative Design: Retrospective cohort Methods and Data: Medicare claims data from 2010–2014. Setting: Nationwide, USA Sample/population characteristics: AWV cohort n = 66,399; control n = 66,399; adults 65+	Outcome measure: Cognitive screening rates, lab tests, dementia medication prescriptions. Intervention: Routine AWV vs. standard care	Embedded lab testing, EHR triggers	Limited provider follow-through. Workflow constraints
Fowler et al., 2020 [13]	To evaluate health-related quality of life impacts of AWV cognitive screening interventions (CHOICE trial).	Study type: Quantitative Design: Randomized control trial. Methods and Data: Medicare claims data from 2010–2014. Setting: Nationwide primary care settings, USA Sample/population characteristics: 3416 older adults (mean age 74.1, diverse racial background).	Outcome measure: Health-related quality of life (HUI), PHQ-9, GAD-7 Intervention: CHOICE trial for ADRD screening during AWVs	Integration of screening into workflows, Participant engagement.	Possible underreporting of challenges with screening sensitivity or equity concerns. Limited diagnostic specificity or generalizability.
Hamer et al., 2023 [30]	To examine the adoption and perceived value of the Medicare AWV among primary care providers and clinics, and to identify factors that influence its delivery.	Study type: Mixed methods study. Design: Convergent mixed-methods study. Methods and Data: Medicare claims data 2015–2018 and semi-structured interviews Setting: National scope Sample/population: primary care practices participating in the National Cancer Institute’s Community Oncology Research Program (NCORP).	Outcome measure: included rates of AWV, provider and clinic perceptions of AWV benefits and barriers, as well as workflow adaptations and implementation support. Intervention: Delivery of the Medicare AWV with structured elements like health risk assessments and cognitive screening.	EHR prompts and templates. Practice-level champions. Organizational support for preventive care. Alignment with value-based care models.	Workflow interruptions. Low perceived value among some providers. Lack of clarity around billing and cognitive assessment expectations. Competing demands in primary care.
Harrison et al., 2024 [17]	To examine the implementation of the PRO-CS Cognitive Function Screener within Medicare AWVs, comparing different framings of cognitive function—framed as “abilities” vs. “concerns.”	Study type: Quantitative Design: Health system implementation evaluation. Methods and Data: PRO-CS implementation analysis; patient-reported outcome data collection via electronic medical record systems. Setting: Pennsylvania. Sample/population: Medicare beneficiaries undergoing AWVs; exact sample not specified.	Outcome Measures: PRO-CS uptake, framing preference, engagement levels. Intervention: Integration of PRO-CS during AWV, comparing ability-focused vs. concern-focused language.	Framing cognitive health as “abilities” improved patient engagement. PRO-CS tools supported integration into electronic workflows.	Framing cognitive function as “concerns” was associated with discomfort and reduced participation. Limited provider confidence in interpreting PRO-CS results
He et al., 2023 [18]	To assess the delivery of high-value services, including AWVs, in Medicare’s advanced primary care models.	Study type: Quantitative. Design: Retrospective cohort analysis. Methods and Data: Medicare claims analysis of high-value service use in primary care models. Setting: Nationwide. Sample/population: National Medicare beneficiary sample; advanced primary care participants vs. non-participants.	Outcome Measures: Use of AWV, preventive screenings, chronic care management. Intervention: Participation in the advanced primary care model.	Organizational incentives tied to care quality. Better infrastructure and leadership support in advanced primary care settings.	Limited scalability beyond pilot or high-resource settings Possible selection bias in participating practices.
Jacobson et al., 2020 [14]	To examine self-reported rates of AWVs and structured cognitive assessments among Medicare Advantage vs. Fee-for-Service enrollees.	Study type: Quantitative. Design: Cross-sectional survey. Methods and Data: Online survey from Understanding America Study panel. Setting: Nationwide. Sample/population: 65+ Medicare beneficiaries; comparison across plan types.	Outcome Measures: Rates of AWV receipt and cognitive screening. Intervention: AWV completion; cognitive screening (structured vs. informal).	Medicare Advantage enrollees had higher AWV and cognitive screening rates Financial and organizational incentives in Medicare Advantage plans.	Fee-for-Service participants had lower uptake and limited access to structured screening tools. Inconsistent application of cognitive screening practices
JaKa et al., 2024 [19]	To evaluate acceptability and feasibility of integrating a cognitive screening tool into Medicare AWVs in clinical workflows, with a focus on dementia-friendly practices.	Study type: Mixed Design: Mixed method hybrid research Methods and Data: Survey and interviews; family medicine practices. Setting: primary care clinics. Sample/population characteristics: 65 providers and 58 patients.	Outcome measures: Perceived feasibility and acceptability of cognitive screening tools Intervention: Explored impact of cognitive assessment during AWV	Provider-patient trust, tool clarity.	Time, discomfort. Supported embedding screening in AWVs.
Johnston et al., 2023 [20]	To assess how participation in ACO models affects AWV access and utilization for older adults with ADRD.	Study type: Quantitative Design: Observational Methods and Data: National claims and Medicare data analysis Setting: Nationwide data Sample/population characteristics: National cohort of Medicare ACOs; older adults with ADRD.	Outcome measure; AWV uptake in dementia populations across ACO types Intervention: Use of ACO structures to influence AWV access and utilization	Organizational support, infrastructure from ACO	Equity and access challenges for vulnerable populations
Jørgensen et al., 2020 [15]	To assess the association between AWV receipt and influenza vaccination rates among older adults	Study type: Quantitative. Design: Retrospective cohort analysis. Methods and Data: Medicare claims data analysis. Setting: Nationwide. Medicare beneficiaries aged 65+, nationwide.	Outcome Measures: Influenza vaccination rates post-AWV. Intervention: Receipt of AWV vs. non-receipt	AWV associated with increased vaccination uptake Preventive care bundling supported follow-through	Some disparities in AWV access (e.g., racial/ethnic minorities). Fragmented follow-up or documentation challenges
Lind et al., 2021 [21]	To evaluate the relationship between direct cognitive assessment introduced with the Medicare AWV and new diagnoses of dementia, and to determine if effects vary by race.	Study type: Quantitative Design: Discrete-time survival analysis. Methods and Data: Medicare claims 2003 to 2014. Setting: Nationwide Sample/population characteristics: 324,485 Fee-for-Service Medicare beneficiaries aged 65+.	Outcome measures: Dementia incidence Intervention: AWV identified via claims code G0402, stratified by race/ethnicity	Annual Wellness Visit utilization was associated with an increased probability of new dementia diagnosis with effects varying by racial group (categorized as white, black, Hispanic/Latino, or Asian based on Social Security Administration data). Early AWV adoption, county-level utilization Dementia diagnosis rates increased with AWV implementation with heterogenous effects by race and ethnicity.	Racial disparities in AWV use
Little et al., 2021 [22]	To explore the integration of AWVs into nursing home resident care and its effect on personalized prevention planning.	Study type: Quantitative. Design: Descriptive cohort. Methods and Data: Nursing home EHR reviews. Setting: St Louis, Missouri. Sample/population: 65+ Nursing home residents receiving AWVs.	Outcome Measures: Personalized prevention plan use, interprofessional team engagement. Intervention: AWV integration into routine nursing home care.	Personalized prevention plans encouraged team coordination Enhanced interprofessional collaboration (e.g., immunizations, fall prevention).	None noted.
Liu et al., 2024 [23]	To assess uptake of AWVs and use of cognitive screening tools, focusing on factors associated with utilization among rural vs. urban populations.	Study type: Quantitative Design: Nationally representative survey study. Methods and Data: Internet-based survey using Understanding America Study (UAS) panel; U.S.-wide data collection. Setting: Nationwide, Sample/population characteristics: N = 1871 Medicare beneficiaries aged 65+, two-thirds aged 70+, 20% racial/ethnic minorities, 29% rural residents.	Outcome measures: Uptake of AWV; Use of cognitive assessments (formal test vs. clinician questions); Predictors of use Intervention: AWV, cognitive assessment conducted via test or inquiry	Having a usual source of care. Rural residency possibly promoting inquiry on memory issues. Perception of AWV as primary setting for cognitive assessment	Time constraints, Clinician confidence, Lack of access to specialists, no separate billing code for brief tests, Uncertainty about screening utility
Masuda et al., 2022 [24]	To evaluate telehealth-facilitated AWV delivery during the COVID-19 pandemic	Study type: Mixed method. Design: Implementation-focused evaluation. Methods and Data: Patient surveys and clinical data. Setting: Central O‘ahu, Hawai’i. Sample/population: Older adults telehealth-eligible Medicare recipients.	Outcome Measures: Patient satisfaction, telehealth access, AWV completion rates. Intervention: AWV delivery via telehealth.	Increased access for patients with mobility limitations. High satisfaction among digitally literate participants.	Digital divide (access, literacy, broadband). Lack of integration with in-person follow-up for complex needs.
Misra et al., 2019 [16]	To assess the effect of AWV receipt on healthcare utilization and Medicare spending over 12 and 24 months, adjusting for the healthy user effect.	Study type: Quantitative Design: Retrospective observational design with propensity score matching. Methods and Data: Medicare claims 2013 to 2017. Setting: Nationwide Sample/population characteristics: 28,053 AWV users vs. 228,053 matched nonusers, Medicare Fee-for-Service beneficiaries.	Outcome measures: Hospital-related utilization, ED visits, Medicare spending Intervention: Standard AWV with exclusion of prior-year users to simulate new-user design.	Reduced inpatient utilization and spending	Residual unobserved confounders (e.g., healthy user effect). No change in hospital use
Nothelle et al., 2021 [25]	To examine the prevalence of positive geriatric screenings during AWVs, the corresponding follow-up actions, and changes in ACP among older adults receiving two AWVs.	Study type: Quantitative Design: Retrospective cohort. Methods and Data: EMR and AWV questionnaire review. Setting/Sample: Older adults (unspecified sample size), community-based primary care, based in Maryland, Virginia, and Washington.	Outcome measures: Positive screens for geriatric syndromes, ACP uptake. Intervention: Standard AWV delivery with follow-up visits.	ACP follow-up ACP more likely at second AWV if done during first	Limited impact on prescribing despite positive screens
Park et al., 2024 [26]	To examine the determinants and effectiveness of having an AWV among Medicare beneficiaries in 2020.	Study type: Quantitative Design: Cross-sectional study. Methods and Data: Medicare Current Beneficiary Survey 2020. Setting: Nationwide Sample/population characteristics: 5840 Medicare beneficiaries, nationally representative.	Outcome measures: Preventive care use, health status, care satisfaction. Intervention: AWV as independent and dependent variable during COVID-19.	Usual source of care. Medicare Advantage enrollment. Being non-Hispanic Black, and, Being Hispanic Having an AWV was associated with increases in preventive care use (COVID vaccine, flu shot, pneumonia shot, and blood pressure measurement)	Minority race/ethnicity, non-metro residence No improvement in satisfaction or health status (although it enhanced preventive care).
Powell et al., 2024 [27]	To explore shared decision-making in AWVs for older adults with and without cognitive impairment.	Study type: Quantitative; Design: EMR data for Medicare Beneficiaries with AWV 2017 to 2022. Methods and Data: AWV documentation analysis, cognitive testing, decision-making quality measures. Setting: nationwide. Sample/population: Older adults receiving AWV across clinical sites.	Outcome Measures: Quality of shared decision-making, cognitive assessment documentation. Intervention: Structured shared decision-making prompts during AWVs.	Prompts improved provider-patient communication Training boosted confidence in engaging patients	Variability in clinician comfort with shared decision-making. Time pressures during AWVs
Smith et al., 2022 [28]	To explore stakeholder and patient-family dyad perceptions of integrating SHARING Choices into the AWV, focusing on ACP facilitation.	Study type: Qualitative Design: Descriptive qualitative. Methods and Data: Semi-structured interviews and focus group. Setting: primary care practices in Baltimore-Washington Sample/population characteristics: 22 patient-family dyads (14 with cognitive impairment); 30 stakeholders (clinicians, staff, administrators).	Outcome measure: Receptivity, barriers, facilitators to SHARING Choices; ACP discussions in AWVs. Intervention: SHARING Choices intervention integrated into AWV with mailing materials, checklist, portal access, ACP facilitator	Relative advantage, adaptability, established training, structured support for ACP	Workflow complexity, spatial constraints, variability in documentation
Tzeng et al., 2022 [29]	To explore the effect of the utilization of AWVs in 2017 on fall and fracture prevention through 31 December 2018.	Study type: Quantitative Design: Retrospective cohort. Methods and Data: Texas Medicare claims data 2014 to 2018. Setting: Texas community dwelling older adults. Sample/population: 1,153,744 Medicare beneficiaries aged 68 years (pre-matching); 742,494 post propensity-score matching. Predominantly female, White, urban residents.	Outcome measures: Incidence of falls and fractures post-AWV (Kaplan-Meier and Cox hazard models) Intervention: Receipt of AWV in 2017; comparisons made with non-AWV recipients over 24-month follow-up	AWV associated with 3.9% reduced risk of falls, 4% reduced risk of fractures, stronger effects for rural residents and those with comorbidities. Further risk reduction for future falls with the receipt of an AWVs in 3 consecutive years	Lower uptake among those with ADRD. Lack of data on AWV delivery process.

Abbreviations: ACOs: Accountable Care Organizations; ACP: Advance Care Planning; ADRD: Alzheimer’s disease and related dementias; AWV: Annual Wellness Visit; EHR: Electronic Health Record; PRO-CS: PROMIS Cognitive Function Screener.

**Table 4:** CFIR-based synthesis to implement AWVs for older adults with cognitive function. a. Facilitators to implementing AWVs. b. Barriers to implementing AWVs.

(a) Facilitators				
CFIR Domain	Theme	Subtheme	Description/Evidence	Citation
Intervention Characteristics	Perceived usefulness of PRO-CS tools	Facilitates early detection and conversation about cognitive issues	Providers found tools like PRO-CS helpful in facilitating discussions on cognition; patients preferred certain formats (e.g., PRO-CS Concerns).	[17]
	Adaptability of AWV model	Integration of AWV into various structured care settings	AWVs adapted well in settings like nursing homes and CPC+ sites, showing flexibility in various care environments.	[18,22]
	Relative advantage over ad hoc visits	AWVs offer structured opportunities for prevention and early detection	Compared to unstructured visits, AWVs allowed for more consistent medication reconciliation and dementia risk screening.	[18,29]
Outer Setting	Policy and program incentives	Participation in CPC+, ACOs, or Medicare Advantage increased AWV use	Policy incentives and value-based payment models improved AWV uptake through bundled care and performance-aligned reimbursement.	[2,14,18,20,23,29]
	Policy and program incentives	Alignment with value-based care	Clinics participating in value-based programs (e.g., ACOs) adopted AWVs more readily due to reimbursement incentives.	[30]
Inner Setting	Organizational integration	Embedded tools and standard forms in EHRs improve AWV delivery	Standardized forms and EHR integration improved workflow efficiency and documentation.	[22]
		Workflow alignment and EHR integration	Clinics with structured EHR templates, clear delegation, and workflow support saw smoother AWV delivery.	[30]
	Leadership and Culture	Quality improvement culture and leadership buy-in	Clinics with strong leadership engagement and a culture of continuous quality improvement were more likely to embed AWVs successfully.	[22,27]
		AWV champions and leadership support	Sites with clinical champions and leadership buy-in demonstrated higher implementation success	[30]
	Role Assignment	Dedicated AWV roles (nurse leads, care coordinators)	Assignment of specific team members to manage AWV tasks improved clinic efficiency and sustainability.	[25]
	Technological Alignment	Integration of PRO-CS into EHR systems	Technological facilitation through tools like PRO-CS improved screening rates and provider engagement.	[17]
Characteristics of Individuals	Provider confidence and acceptance	Positive attitudes towards cognitive screening	Providers supported the use of PRO-CS and recognized the importance of cognitive screening.	[17]
		Impact of training and familiarity with tools	Provider confidence was strengthened through exposure and training in tools like the PRO-CS, increasing comfort in addressing cognitive concerns.	[17]
	Communication approach	Strengths-based communication improves engagement	Framing cognitive assessments using strengths-based language (e.g., “abilities” rather than “deficits”) improved patient receptivity and provider-patient dialogue.	[17]
	Individual readiness	Belief in AWV value	Providers who believed in AWVs as tools for early detection and planning were more likely to perform them consistently and with fidelity.	[21,26]
		Intrinsic motivation and professional identity	Providers with a geriatric focus or experience with ADRD were more likely to fully implement AWVs and follow through with referrals and care planning.	[23,25]
Implementation Process	Structured implementation strategies	Use of interprofessional teams and regular follow-ups	Nursing homes that implemented structured AWV visits through interprofessional teams showed improvement in care indicators.	[22]
	Structured implementation strategies	Use of interprofessional teams and regular follow-ups	Nursing homes that implemented structured AWV visits through interprofessional teams showed improvement in care indicators.	[22]
	Technological aids improve fidelity	EHR-embedded templates, prompts, and checklists	Embedding cognitive screening tools and AWV templates into EHRs improved workflow consistency and screening completion.	[17,23,30]
	Staff preparation enhances workflow	Pre-visit planning and staff training	Training staff to conduct pre-visit chart reviews, gather baseline data, and prepare patients enhanced AWV flow and consistency.	[27,28]
	Continuous refinement improves sustainability	Use of iterative Quality Improvement strategies (e.g., PDSA cycles)	Sites employing PDSA cycles and feedback loops refined AWV implementation over time and improved adoption rates.	[27]
**(b) Barriers**				
**CFIR Domain**	**Theme**	**Subtheme**	**Description/Evidence**	**Citation**
Intervention Characteristics	Clinical Effectiveness and Utility	Inconsistent impact of AWVs on ADRD diagnosis and management	No clinically meaningful differences found in ADRD diagnoses or initiation of dementia-specific medications between AWV and control groups, raising concerns about clinical effectiveness.	[12]
	Clinical Effectiveness and Utility	Perceived burden and complexity	The time-intensive nature and unclear clinical benefit of AWVs—especially for cognitive components—led to low uptake in some practices.	[17,27]
	Design and Delivery Barriers	Ambiguity in cognitive screening requirements	Providers reported confusion about what qualifies as a “structured” cognitive assessment, limiting fidelity and consistency.	[19]
Outer Setting	Patient-level challenges	Cognitive impairment, multimorbidity, and functional limitations hinder AWV participation	High prevalence of cognitive impairment and functional decline among older adults reduces AWV uptake, particularly in nursing homes and underserved populations.	[15,22]
	Structural and policy gaps	Lack of clear implementation guidance for cognitive screening	AWVs are underutilized for dementia screening due to unclear CMS policies and limited reimbursement for cognitive assessment tools.	[14]
	Health equity and access gaps	Geographic and demographic disparities in AWV delivery	Uptake was lower among underserved groups due to structural barriers and access issues.	[2,16,23,26]
	Patient engagement barriers	Low patient awareness and competing priorities	In disadvantaged settings, AWVs were deprioritized due to other health and social needs.	[12,20]
Inner Setting	Workflow and resource constraints	Limited staff time, EHR fragmentation, and competing priorities	Clinics report difficulty incorporating AWVs due to staffing shortages, EHR integration issues, and high patient volumes.	[13]
		Competing demands and workflow interruptions	Practices reported that AWVs were disrupted by competing clinical demands and poorly integrated workflows.	[30]
	Training and staff preparedness	Inadequate training on AWV and cognitive components	Lack of staff awareness and training—especially around cognitive screening—led to inconsistent delivery and low confidence in implementation.	[23]
	Organizational alignment	AWVs deprioritized due to competing care demands	AWV implementation often conflicted with organizational priorities emphasizing productivity or acute care, limiting time and support for preventive visits.	[19]
	Workflow burden	Misalignment with routine visit structure	AWVs were perceived as time-consuming and disruptive to standard visit workflows, requiring extra appointments and preparation that strained clinic capacity.	[24]
Characteristics of Individuals	Provider hesitation	Discomfort with cognitive screening or interpreting results	Some providers express concern about the reliability of patient-reported cognitive assessments and lack of training on interpreting results.	[17]
		Low perceived value of AWVs by some providers	Some providers questioned the utility of AWVs, especially in cognitively impaired patients, which affected engagement and follow-through.	[30]
	Communication confidence	Discomfort initiating sensitive conversations	Providers reported hesitancy in discussing cognitive decline due to fear of causing distress or lack of conversational strategies.	[27]
	Competing clinical priorities	AWVs deprioritized during acute care demands	Time pressures and the need to manage acute conditions often led providers to delay or skip AWVs despite eligibility.	[20]
Implementation Process	Inconsistent or absent implementation protocols	Lack of structured workflows, follow-up pathways, or leadership engagement	Sites without formal AWV programs or guidance struggle to systematically deliver AWVs and integrate findings into patient care.	[13,18]
		Lack of clarity around AWV components.	Clinics lacked standardized processes for cognitive assessment and billing, leading to inconsistent implementation.	[30]
	Performance Monitoring Deficits	No systematic tracking of AWV or dementia indicators	Lack of performance feedback limited continuous improvement and accountability.	[16,20]
	Weak Leadership Engagement	AWVs not prioritized by clinic leadership	In the absence of leadership support, AWVs were often deprioritized or viewed as burdensome.	[13,18,29]
		Limited prioritization of preventive care	AWVs were often not embedded as a strategic or leadership priority, reducing resource allocation and organizational alignment.	[30]

Footnote: Barriers and facilitators listed in Table 4 were synthesized across all included studies and were not stratified by care setting. Included studies represent diverse settings such as primary care clinics, specialty geriatric practices, large healthcare systems, and long-term care facilities. As such, implementation insights reflect system-level patterns and cross-setting dynamics rather than setting (or context)-specific conclusions. Abbreviations: ACOs: Accountable Care Organizations; ADRD: Alzheimer’s disease and related dementias; AWV: Annual Wellness Visit; CPC+: Comprehensive Primary Care Plus; CMS: Centers for Medicare and Medicaid Services; EHR: Electronic Health Record; PRO-CS: PROMIS Cognitive Function Screener.

## Data Availability

No new data were created. Readers may request the corresponding author’s summary information in the tables via email.

## Public Involvement Statement

No public involvement in any aspect of this research. Guidelines and Standards Statement: This manuscript was drafted according to the guidelines of the Preferred Reporting Items for Systematic Reviews and Meta-Analyses Extension for Scoping Reviews (PRISMA-SCR) [10].

## Guidelines and Standard Statement

This manuscript was drafted against the recommendations of the Preferred Reporting Items for Systematic Reviews and Meta-Analyses Extension for Scoping Reviews.

## Abbreviations

The following abbreviations are used in this manuscript:

ACOs	Accountable Care Organizations
ACP	Advance Care Planning
ADRD	Alzheimer’s disease and related dementias
AWV	Annual Wellness Visit
CFIR	Consolidated Framework for Implementation Research
CMS	Centers for Medicare and Medicaid Services
EHR	Electronic Health Record
FTAs	full-text articles
GUIDE	Guiding an Improved Dementia Experience
MCI	mild cognitive impairment
MeSH	Medical Subject Headings
PRISMA-SCR	Preferred Reporting Items for Systematic Reviews and Meta-Analyses Extension for Scoping Reviews
PRO-CS	PROMIS Cognitive Function Screener

## Appendix A. Critical Appraisal of the Included Analytical Cross-Sectional Studies Using the Joanna Briggs Institute Critical Appraisal Tools

	**Cross-Sectional Study**	**Jacobson, Thunell, & Zissimopoulos, 2020 [14]**	**Jørgensen et al., 2020 [15]**	**Park & Nguyen, 2024 [26]**
1	Were the criteria for inclusion in the sample clearly defined?	Y	Y	Y
2	Were the study subjects and the setting described in detail?	Y	Y	Y
3	Was the exposure measured in a valid and reliable way?	Y	Y	Y
4	Were objective, standard criteria used for measurement of the condition?	Y	Y	Y
5	Were confounding factors identified?	N	N	N
6	Were strategies to deal with confounding factors stated?	N	N	N
7	Were the outcomes measured in a valid and reliable way?	Y	Y	Y
8	Was appropriate statistical analysis used?	Y	Y	Y
Key: Y = Yes; N = No.				

## Appendix B. Critical Appraisal of the Included Cohort Study Research Using the Joanna Briggs Institute Critical Appraisal Tools

	**Cohort Studies**	**Fowler et al., 2018 [12]**	**He et al., 2023 [18]**	**Lind et al., 2021 [21]**	**Misra & Lloyd, 2019 [16]**	**Johnston et al., 2023 [20]**	**Little et al., 2021 [22]**	**Tzeng et al., 2022 [29]**
1	Were the two groups similar and recruited from the same population?	Y	Y	Y	Y	Y	Y	Y
2	Were the exposures measured similarly to assign people to both exposed and unexposed groups?	Y	Y	Y	Y	Y	Y	Y
3	Was the exposure measured in a valid and reliable way?	Y	Y	Y	Y	Y	Y	Y
4	Were confounding factors identified?	Y	U	Y	Y	Y	Y	Y
5	Were strategies to deal with confounding factors stated?	Y	U	Y	Y	Y	Y	Y
6	Were the groups/participants free of the outcome at the start of the study (or at the moment of exposure)?	Y	Y	Y	U	Y	Y	Y
7	Were the outcomes measured in a valid and reliable way?	Y	Y	Y	Y	Y	Y	Y
8	Was the follow up time reported and sufficient to be long enough for outcomes to occur?	Y	Y	Y	Y	Y	Y	Y
9	Was the follow up complete, and if not, were the reasons to loss to follow up described and explored?	Y	Y	Y	Y	Y	Y	Y
10	Were strategies to address incomplete follow up utilized?	NA	NA	NA	U	NA	NA	NA
11	Was appropriate statistical analysis used?	Y	Y	Y	Y	Y	Y	Y
Key: Y = Yes; U = Unclear; NA = Not applicable.								

	**Cohort Studies**	**Nothelle et al., 2022 [25]**	**Powell et al., 2024 [27]**	**Smith et al., 2022 [28]**	**Liu et al., 2025 [23]**
1	Were the two groups similar and recruited from the same population?	Y	Y	Y	Y
2	Were the exposures measured similarly to assign people to both exposed and unexposed groups?	Y	Y	Y	Y
3	Was the exposure measured in a valid and reliable way?	Y	Y	Y	Y
4	Were confounding factors identified?	Y	Y	Y	Y
5	Were strategies to deal with confounding factors stated?	Y	Y	Y	Y
6	Were the groups/participants free of the outcome at the start of the study (or at the moment of exposure)?	Y	Y	Y	Y
7	Were the outcomes measured in a valid and reliable way?	Y	Y	Y	Y
8	Was the follow up time reported and sufficient to be long enough for outcomes to occur?	Y	Y	Y	Y
9	Was the follow up complete, and if not, were the reasons to loss to follow up described and explored?	Y	Y	Y	Y
10	Were strategies to address incomplete follow up utilized?	NA	NA	NA	NA
11	Was appropriate statistical analysis used?	Y	Y	Y	Y
Key: Y = Yes; NA = Not applicable.					

## Appendix C. Critical Appraisal of the Included Studies Using a Quasi-Experimental Study Design (Using an Evaluation Study Design) Using the Joanna Briggs Institute Critical Appraisal Tools

	**Quasi-Experimental Studies (Using an Evaluation Study Design)**	**Fowler et al., 2020 [13]**	**Harrison et al., 2024 [17]**
1	Is it clear in the study what is the ‘cause’ and what is the ‘effect’ (i.e., there is no confusion about which variable comes first)?	Y	Y
2	Were the participants included in any comparisons similar?	Y	Y
3	Were the participants included in any comparisons receiving similar treatment/care, other than the exposure or intervention of interest?	N	N
4	Was there a control group?	N	N
5	Were there multiple measurements of the outcome both pre and post the intervention/exposure?	N	N
6	Was follow up complete and if not, were differences between groups in terms of their follow up adequately described and analyzed?	Y	Y
7	Were the outcomes of participants included in any comparisons measured in the same way?	Y	Y
8	Were outcomes measured in a reliable way?	Y	Y
9	Was appropriate statistical analysis used?	Y	Y
Key: Y = Yes; N = No.			

## Appendix D. Critical Appraisal of the Included Studies Using a Quasi-Experimental Study Design (A Mixed Methods Study Design) Using the Joanna Briggs Institute Critical Appraisal Tools

	**Mixed Methods Studies (Using a Mixed Methods Study Design)**	**Hamer et al., 2023 [30]**	**JaKa et al., 2024 [19]**	**Masuda et al., 2022 [24]**
1	Were the inclusion criteria clearly defined?	Y	Y	Y
2	Were the study subjects and setting described in detail?	Y	Y	Y
3	Was the exposure measured in a valid and reliable way?	NA	Y	Y
4	Were objective, standard criteria used to measure the condition?	Y	Y	Y
5	Were confounding factors identified?	NA	Y	NA
6	Were strategies to deal with confounding factors stated?	N	Y	Y
7	Were the outcomes measured in a valid and reliable way?	Y	Y	Y
8	Were appropriate statistical analyses used?	Y	Y	Y
Key: Y = Yes; NA = Not applicable.				
